# 11th international multithematic scientific biomedical congress (IMBMC), Nicosia, Cyprus, 2023

**DOI:** 10.1038/s41419-024-06623-8

**Published:** 2024-04-17

**Authors:** Panayiota Christodoulou, Maria-Areti Salamouri, Ioannis Papavasileiou, Theodora-Christina Kyriakou, Charalambos Michaeloudes, Petros Agathaggelou, Anastasis Stephanou, Ioannis Patrikios

**Affiliations:** 1 School of Medicine, European University Cyprus, Nicosia, Cyprus; 2 School of Dentistry, European University Cyprus, Nicosia, Cyprus

**Keywords:** Epidemiology, Physiology

The 11th international multithematic biomedical congress (IMBMC) 2023, “Bio-Medical Scientific Cyprus,” took place at European University Cyprus (EUC), Nicosia, Cyprus, under the auspices of the Ministry of Health and the Cyprus Medical Association. IMBMC is an internationally-recognized event, founded and established by Prof. Dr. Ioannis Patrikios, the Deputy Dean of the School of Medicine at EUC. During the 11th IMBMC, Prof. Dr. Harvey J. Alter (Nobel prize award in medicine, in 2020, discovery of hepatitis C virus) was honored with the title of Honorary Professor of the School of Medicine, European University, Cyprus and Prof. Dr. Joseph Brugada Terradellas (discovery of Brugada Syndrome) received a Doctor Honoris Causa Degree.

**Dr. John Ioannides**, Professor of Medicine, Epidemiology and Biomedical Data Science and Statistics, presented the topic “Revisiting COVID-19 epidemiology after pandemic,” focusing on improving research methodologies and refining the ability to generate dependable evidence by combining information. His lecture was focused on the COVID-19 pandemic since 2019 and covered epidemiology, modeling, risk stratification, effective and ineffective interventions, policies, benefits and harms of different approaches. Finally, he discussed unresolved controversies that need to be evaluated following the peak crisis period.

**Dr. Philip Calder**, Professor of Nutritional Immunology and Head of the School of Human Development and Health, Faculty of Medicine, University of Southampton, lectured on “Pleiotropic effects of PUFAs explain their role in cardio protection.” He explained the modulation of the structure and function of cell membranes by polyunsaturated fatty acids (PUFAs), which are precursors of oxylipins that control transcription factors and modulate gene expression. PUFAs were shown to potentially be a useful diagnostic tool for limiting the risks and severity of cardiovascular diseases. The amount of linoleic acid contained in omega-3 PUFAs could reduce LDL cholesterol, through the expression and activating Sterol regulatory-element binding proteins (SREBPs). Consumption of arachidonic acid, an omega-6 PUFA needs to be regulated as it has been shown to affect thrombosis and inflammation by increasing the amounts of oxylipins. Omega-3 PUFAs, eicosapentaenoic acid (EPA) and docosahexaenoic acid (DHA), can decrease the oxylipin levels produced by arachidonic acid, hence lowering the risk of thrombosis and inflammation. Epidemiological studies and clinical trials demonstrated the positive effect of EPA and DHA on multiple risk factors for cardiovascular diseases, such as enhancing the atherosclerotic plaque stability.

**Dr. Kyriakos Kypreos**, Professor and chairman of the Pharmacology Laboratory of the Department of Medicine at the University of Patras School of Health Sciences, Patra, Greece and adjunct Professor of Pharmacology and Metabolic Disorders at the European University Cyprus, School of Medicine, presented the topic entitled “Interconnection between obesity and lipoproteins: mechanisms beyond intuition.” His research showed the effect of the lipoprotein transport system on regulating dietary and endogenously-synthesized lipids. Atherogenic lipoproteins could potentially induce white adipose tissue (WAT) and brown adipose tissue (BAT) mitochondrial metabolic dysfunction, while HDL could act as a protective factor. Peripherally-expressed Apolipoprotein E (APOE) was previously thought to induce obesity by delivering receptor-mediated postprandial lipids to WAT. However, peripherally-expressed APOE3 was shown to switch substrate oxidation towards non-shivering thermogenesis in visceral WAT mitochondria, preventing obesity. His research work, reveals new mechanisms through which lipoproteins regulate WAT and BAT energy metabolism that can lead to new approaches for obesity management.

**Dr. Stefano Del Prato**, Professor of Endocrinology and Metabolism at the University of Pisa School of Medicine and Chief of the Section of Diabetes, University of Pisa, Italy, an honorary keynote speaker, gave a talk entitled “Modern approaches to treatment of Type 2 diabetes.” His research was focused on the treatment of hyperglycemia in Type 2 diabetes. Clinical trials proved that glucagon-like peptide 1 receptor agonists (GLP1-RAs) could improve both glycemic control and lower the body weight, while simultaneously acting as protective agents against cardiovascular diseases, in type 2 diabetes patients with atherosclerosis or at high-risk of developing atherosclerosis. In combination with these agents, SGLT2-inhibitors were also found to offer protection against cardiovascular disease with a greater benefit on renal function and a specific decrease in the risk of hospitalization due to heart failure. The crucial result was that these effects did not show a difference between diabetic and non-diabetic patients. The microvascular complications should be taken into account as they increase the correlation between overall glycemic exposure and cardiovascular disease risk. Through studies involving large population samples, it was shown that cardiovascular disease burden was affected by glycemic control, LDL-cholesterol and blood pressure.

Identical data have also revealed that individuals managing Type 2 diabetes effectively, by controlling HbA1c, LDL-cholesterol, albuminuria, and blood pressure, while abstaining from smoking, exhibit a cardiovascular risk nearly indistinguishable from those without diabetes. The overall aim of this research is to achieve a more holistic, person-centered approach with a focus on glycemic control, body weight regulation and cardiovascular risk factors.

**Dr. Vasso Apostolopoulos**, Professor, Vice-Chancellor’s Distinguished Professorial Fellow, and Head of Immunology and Translational Research at Victoria University, Australia, gave a lecture on “Identification of cancer pathways and markers in mouse models of spontaneous chronic colitis: From inflammation to cancer.” Her research focused on the key role of chronic inflammation in oncogenesis and the link between inflammatory bowel disease and the development of cancer. By delving deeper into the genetic basis of how inflammatory bowel disease could lead to malignancy, and the potential use of biomarkers for screening the development of cancer. An animal model of chronic colitis was used to understand the expression of genes, including checkpoint markers, cancer-related pathways and cancer genes in colon tissues.

**Dr. Konstantinos Dimas**, Professor at the Department of Pharmacology in School of Medicine of Thessaly, presented his research entitled “A novel rare triple negative breast cancer (TNBC) patient-derived xenograft: Development, characterization, and application.” His research work provided insights regarding breast cancer and the use of xenographs (PDX) acquired from triple-negative/ lipid rich breast cancer (TN/LRBC) patients. His investigation involved immunocompromised mice, which underwent direct transplantation of tumors that were surgically removed from patients. The findings showed that the xenograft had a positive response to cyclophosphamide and docetaxel, while doxorubicin had increasing levels of toxicity. A different pharmacological approach involved Caelyx® (stealth liposomal doxorubicin), which was shown to be highly effective with little toxicity. The innovative PDX for TN/LRBC is a rather promising model that can potentially be a useful source for both the development of new drug therapies and for understanding the biological background of this rare type of breast cancer.

**Dr. Florita Poulakaki**, director of the Breast Clinic in the Athens Medical Center and Vice president Europa Donna, gave a talk on “Advances in Breast Surgery.” The gold-standard screening method for breast cancer is known to be Sentinel Lymph Node Biopsy. This technique achieves successful evaluation of lymph nodes, eliminating the requirement of complete dissection of the axillary lymph node. Because of its minimally-invasive nature, it reduces the risk of post-operational lymphedema. Regarding oncoplastic surgery, the method of Nipple-Sparing Mastectomy (NSM) provides a highly aesthetic outcome as the nipple and areola tissues are preserved. Another novel method includes the use of ADM, a surgical mesh derived from either human or animal tissue, free of cells and left-over support structure which is left inside the breast. Other reconstructions that have achieved a good aesthetic outcome involve autologous tissue flaps and implant-based reconstruction. As far as screening is concerned, both the Genetic Testing Panels and Risk Assessment Molecular profiling may enhance the understanding of breast cancer genetics as she stated. All the scientific advances mentioned improved key parameters of breast surgery, such as increasing the survival rate, better aesthetics, and elimination of scarring.

**Dr. George Paxinos**, Professor at the University of New South Wales, presented the scientific topic entitled “Is the Brain in the Goldilocks Zone?” He proposed that the field of chemoarchitecture could be considered as a tool to identify nuclei and aid in the establishment of homologies between animals and humans. In his clinical study, he compared the human brain to that of primates including species of chimpanzee, rhesus macaque and marmoset and found that the nuclei of all primates were the same. The presentation highlighted the evolution of the human brain structure and the neuroscience principles included in his novel “A River Divided.”

**Dr. Harvey J. Alter**, Professor of Physiology at the National Institute of Health, Bethesda, who was awarded the Nobel prize in medicine for the discovery of the hepatitis C virus in 2020 and had a significant role in the discovery of two hepatitis viruses, in particular hepatitis B virus (HBV) and hepatitis C virus. Dr. Harvey J. Alter conducted various longitudinal studies involving transfusion-associated hepatitis (TAH). The results consecutively concluded that, prior to 1970, TAH had an increased prevalence among open-heart surgery patients, while it was shown that paid donors had seven times greater risk than volunteer donors to transmit TAH. When a system involving solely volunteer donors in combination with initial generation screening for hepatitis B antigen, there was a 70% reduction of TAH. The notion that most TAH cases were neither related to hepatitis A nor hepatitis B viruses, resulted in the assignment of non-A, non-B hepatitis (NANBH). It was further discovered, upon liver biopsy, that NANBH could cause liver cirrhosis and it was then revealed that NANBH was identical to hepatitis C virus (HCV). A significant breakthrough involved the association of HCV with being the predominant cause of hepatocellular carcinoma. In 2014, the introduction of HCV-specific direct antivirals showed a dramatic rate of cure of 95%-100% of chronic HCV in a period within 8-12 weeks without any crucial side effects. This scientific research holds a future promise in relation to the worldwide elimination of HCV infection.

**Dr. Christos Savopoulos**, Professor of Internal Medicine at the Aristotle University of Thessaloniki, presented his research on the “Clinical relevance of adverse remodeling of microcirculation in hypertensive patients, at the level of cerebral circulation.” Dr. Savopoulos and his team gave a review regarding lacunar infarcts and lipohyalinosis under the spectrum of cerebral small vessel disease. Lacunar stroke pathophysiology and classifications, following neuroimaging techniques, were analyzed. The investigation of these common diseases can provide researchers with specific molecular targets which will limit the disease. Summing up, the algorithm may lead to an accurate diagnosis and correct treatment approach of lacunar infarct.

**Dr. Philip Froguel**, Professor and Chairman of Genomic Medicine at Imperial College London, presented on the topic “Precision Diabetes and obesity medicine: achievements and future outcomes.” In 1992, Professor Froguel identified glucokinase, the first gene implicated in the pathogenesis of diabetes. In 1998, he investigated the primary factor of monogenic obesity (MC4R) and identified the first recessive mutation in the leptin receptor gene, which causes obesity. In 2006, he discovered the impact of the sulfonylurea gene ABCC8 in monogenic diabetes, and in 2012-2011 he reported that Copy Number Variation leads to extreme obesity or leanness depending on the DNA quantity. His publication of Genome Wide Association Study (GWAS) in Type 2 Diabetes (T2D) included the discovery of the first gene for regular obesity (FTO). In 2007, he discovered the first gene frequent variants regulating glycemia (in GCPC2) and unveiled the involvement of the melatonin pathway as a risk factor for T2D. His most recent findings include the existence of pathogenic mutations in 3% of patients with common T2D, creating an avenue to precision medicine. Presently, Professor Froguel is engaged in research into personalized medicine, aiming to pinpoint diabetic patients who would benefit from tailored treatments for disease management and complication prevention.

**Dr. Katerina Naka**, Professor of Cardiology in the Faculty of Medicine, University of Ioannina, spoke on her research on the “Current treatment of Hf- good news for HfpEf.” The primary risk factor for heart failure with preserved ejection fraction (HFpEF) is hypertension. HFpEF syndrome has elevated risk regarding cardiovascular morbidity and mortality. While specific management for HFpEF is not firmly established, due to limited trial data, the control of blood pressure is a crucial preventive factor. Recent guidelines recommend medications that target the renin- angiotensin system (RAS), including ACE inhibitors or angiotensin receptor blockers as a therapy for HFpEF patients. The modified treatment includes the use of spironolactone and the angiotensin receptor neprilysin inhibitor. More recent investigations proposed the use of sodium- glucose co-transporter- 2 inhibitors, currently used in high-risk diabetes patients to prevent heart failure hospitalizations. as a first line therapy for HFpEF, following their proven effectiveness in RCTs. The updated guidelines offer a clearer path for identifying HFpEF phenotypes and treatment planning, addressing the needs of hypertensive patients with HFpEF.

**Dr. Paolo R Madeddu**, Professor and Chairman of Experimental Cardiovascular Medicine at the Bristol Heart Institute, gave a lecture entitled: “Drug repurposing for treatment of cardiac steatosis and ischaemia.” Drug repurposing involves utilizing a medication for purposes different than its originally intended use. The concept is appealing due to its potential to decrease the expenses and time required for developing a new drug. In the realm of cardiovascular medicine, repurposing drugs has often been driven by opportunistic discoveries or observations of how they affect disease mechanisms, rather than following systematic methodologies. Lately, tyrosine kinase inhibitors (TKIs), originally investigated in cancer studies, have found new applications in diabetes treatment. Likewise, MEK1/2 inhibitors, known for their potential in cancer therapy, have demonstrated the ability to enhance elastin synthesis in both laboratory settings and live animal studies. This suggests that inhibiting ERK-1 and -2 could be a potential treatment avenue for vascular conditions characterized by decreased arterial elastin levels. Blocking MEK activity also exhibits antiatherogenic effects. When MEK inhibition is paired with the activation of liver X receptor (LXR), it notably hampers atherosclerosis progression in mice lacking ApoE (ApoE-/-). This combined approach operates through mechanisms such as reverse cholesterol transport and the prevention of foam cell formation, contributing significantly to inhibiting atherosclerosis development. Novel findings were presented indicating that TKIs and ERK1/2 inhibitors hold promise in addressing cardiac steatosis and myocardial ischaemia. Additionally, fresh methodologies were introduced for systematically screening drugs aimed at identifying their potential applications in cardiovascular treatments.

**Prof. Josep Brugada Terradellas**, MD, PhD, FESC, an honorable keynote speaker, is a cardiologist, specialist in biology and sport medicine specialist. He discovered the Brugada syndrome in 1992. The initial documentation of the Brugada syndrome described it as a hereditary condition marked by ST segment elevation in the right precordial within the heart with no structural abnormalities. This elevation often leads to ventricular fibrillation, posing a heightened risk of sudden cardiac death. Presently, clinical diagnosis relies on identifying a specific electrocardiogram pattern, either occurring spontaneously or induced via a pharmacological test. The syndrome’s prevalence varies across different regions and ethnic groups, predominantly impacting adult males. Genetic alterations, particularly within the SCN5A gene, are deemed the primary cause, although several other genes have been linked to the condition. Despite comprehensive genetic analysis, discernible genetic alterations are only identified in about 35% of families. Managing patients and accurately gauging risk, particularly in asymptomatic individuals, presents significant challenges. The most efficacious treatment remains the implantation of a cardiac defibrillator, while recent reports suggest radiofrequency ablation as a potentially promising novel therapeutic approach.

